# Impact of metabolic syndrome on the risk of endometrial cancer and the role of lifestyle in prevention

**DOI:** 10.17305/bjbms.2021.6963

**Published:** 2022-02-27

**Authors:** Alejandra Rocío Pérez-Martín, Denisse Castro-Eguiluz, Lucely Cetina-Pérez, Yadira Velasco-Torres, Antonio Bahena-González, Edgar Montes-Servín, Ernesto González-Ibarra, Raquel Espinosa-Romero, Dolores Gallardo-Rincón

**Affiliations:** 1 Ovarian and Endometrial Cancer Program (COE), Instituto Nacional de Cancerología (INCan), Mexico City, Mexico; 2 Investigador por México CONACYT-Department of Clinical Research, Instituto Nacional de Cancerología (INCan), Mexico City, Mexico; 3 Department of Clinical Research and Medical Oncology, Instituto Nacional de Cancerología (INCan), Mexico City, Mexico; 4 Department of Medical Oncology, Instituto Nacional de Cancerología (INCan), Mexico City, Mexico

**Keywords:** Metabolic syndrome, endometrial cancer, obesity, diet, prevention strategies

## Abstract

Endometrial cancer (EC) is the second gynecological cancer with the highest global incidence. Among many associated risk factors, metabolic syndrome (MetS) is an important and preventable one. It comprises a group of conditions that often occur together: central adiposity, hyperglycemia, arterial hypertension, and atherogenic dyslipidemia. This review aimed to describe the epidemiological and biological relationship between MetS and EC, focusing on the role of lifestyle in prevention. A literature search was carried out in the PubMed database. 4824 publications were screened, and 123 were included for this review. The association between MetS and EC has been described. Chronic adipose tissue inflammation and insulin resistance are involved in the development of obesity, particularly visceral adiposity. These changes promote the ideal environment for the development of EC. Strategies based on lifestyle modifications may be effective for the prevention of MetS and consequently EC. Some of these modifications include adopting a diet rich in fruits, vegetables, whole grains, and legumes, depending to the accessibility of these foods for each region. Avoiding ultra-processed foods and increasing daily physical activity were also some suggested modifications. We propose that women be screened for MetS to establish early treatment and to possibly prevent EC. Clinical trials designed to prove the effect of lifestyle modifications on the prevention of EC are needed.

## INTRODUCTION

Endometrial cancer (EC) ranks second with the highest global incidence according to the most recent data from GLOBOCAN, 417,000 new cases and 97,000 deaths were estimated in 2020 [1]. In Mexico, EC is the second most frequent gynecological cancer [2], with 5508 new cases and mortality of 1164 cases [3].

Risk factors for developing EC include endocrine factors, such as the use of tamoxifen (relative risk [RR], 2.29) [4], unopposed estrogen therapy (RR, 2.3) [5], polycystic ovary syndrome (odds ratio [OR], 2.79) [6]; gynecological factors such as early menarche [7], nulliparity, anovulation [8], and late menopause (RR 1.89) [9]; obesity (RR 2.21) [10]; diabetes (RR 1.72) [11]; arterial hypertension (AH), (RR 1.61) [12]; metabolic syndrome (MetS) (RR 1.89) [10]; and genetic factors like Lynch syndrome [8].

EC is more commonly diagnosed after menopause and after 60 years of age. However, obesity is related to the diagnosis of EC at an earlier age, particularly the endometrioid subtype [13,14].

In 1983, Bokhman proposed a classification into two clinicopathological types of EC. Type I tumors comprise the majority of EC (80-90%) [15]. These tumors are frequently found in obese women and usually characterized by endometrioid subtype. They are associated with hyperestrogenism due to high body mass index (BMI), and often type 2 diabetes mellitus (T2DM), AH [16], or visceral obesity, and arise from endometrial hyperplasia. Patients with grade 1 endometrioid adenocarcinoma generally have a good prognosis when diagnosed at early stages [8].

In contrast, type II tumors are frequently found in non-obese women, characterized by non-endometrioid subtypes (serous, clear cell, undifferentiated carcinomas, and carcinosarcomas). These are independent of endocrine or metabolic disorders. In addition, type II tumors generally have a worse prognosis than type I tumors [17,18].

This article aims to describe the epidemiological and biological relationship between MetS and EC and the role of lifestyle in prevention.

## MATERIALS AND METHODS

We reviewed articles which focused on the association between MetS and EC, as well the role of lifestyle in prevention. The PubMed database was used for the bibliographic search, restricted by the period 1990-2021. The selection of articles included original articles, review articles, systematic reviews, meta-analysis, conference documents, and scientific statements. Articles published in English and Spanish language were analyzed.

All combinations with the following keywords were included: endometrial cancer, epidemiology, metabolic syndrome, obesity, diabetes, hypertension, biological mechanism, prevention strategies, diet, lifestyle, and physical activity.

The Cochrane systematic reviews database was also searched, using the keywords “metabolic syndrome” and “endometrial cancer” restricted by the period 1990-2021, but no publications were found.

Articles that were focused on pharmacological and surgical treatment were excluded because these were not the aim of this study. Trials that included other types of gynecological cancer were also excluded. A total of 4824 publications were screened, and 123 were included for this review.

## RESULTS AND DISCUSSION

### Metabolic syndrome

MetS is a group of conditions that often arise at the same time, such as central adiposity (high waist circumference, and WC), hyperglycemia, AH, and atherogenic dyslipidemia. MetS increases the risk for coronary heart disease, T2DM, fatty liver, and cancer, including EC [19-22]. Its clinical and epidemiological importance has led various world organizations to establish their criteria to define MetS over time. Table 1 includes the most relevant definitions [19,23-26].

**TABLE 1:**
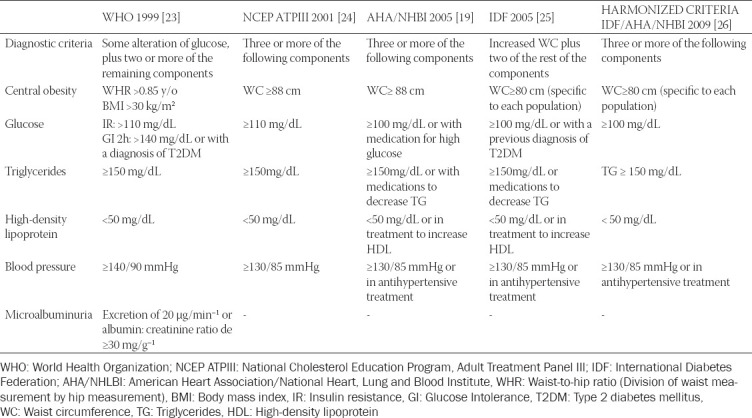
Diagnostic criteria for metabolic syndrome for women worldwide according to different health organizations

The incidence of EC has been rising between 2006 and 2015. It increased on average 1.3% per year in the United States. There is also evidence of increased mortality rates [27]. This phenomenon might be due to the rise of MetS components in women [21,28].

National Health and Nutrition Survey of Mexico (ENSANUT) reported in 2018, a prevalence of 76.8% overweight and obese adult Mexican women [29], the prevalence of abdominal obesity was 88.4%, prevalence of hypertension was 20.9%, and prevalence of T2DM was 11.4% [30]. These statistical data show that large population of Mexican women have at least one MetS component. We hypothesize that this increase may be related to several factors. The Mexican population is genetically predisposed to MetS. Studies have described genetic variants that confer a higher risk for the development of T2DM (*SLC16A11*) and dyslipidemia (*ABCA1/R230C*) in mestizo and indigenous Mexican populations [31]. Environmental factors play an essential role in increasing MetS, such as consumption of a western dietary pattern and sedentary behaviors [32,33].

Alarmingly, sedentarism has increased considerably in the last 2 years because of the COVID-19 pandemic. Due to this, we may expect a significant increase in MetS in the following years.

The relationship of MetS and its components with EC was described by Esposito et al. in a meta-analysis that included six studies and 3132 EC cases. Women with MetS were found to have a RR of 1.89 for developing EC. When analyzing the influence of each MetS component, they found the strongest association for increasing rates in BMI and WC (RR, 2.21). Hyperglycemia and AH were both associated with EC (RR, 1.81 each), and to a lesser extent, high triglyceride (TG) values (RR, 1.17). Low high-density lipoprotein (HDL) levels were not associated with EC [10]. On the other hand, Wang et al. found in a meta-analysis a higher risk of EC in women with MetS (RR, 1.62) [34].

Gutiérrez-Solis et al. carried out a meta-analysis that included 15 reports published between 2004 and 2016 to determine prevalence of MetS in Mexico, and found a prevalence of 31-54%. Each study used different diagnostic criteria for MetS, which accounts for the diversity in results [35]. The prevalence by World Health Organization (WHO) criteria, National Cholesterol Education Program, Adult Tretment Panel III (NCEP ATP III), American Heart Association/National Heart, Lung and Blood Institute (AHA/NHLBI), and International Diabetes Federation (IDF) was 31% [23], 36% [24], 48% [19], and 54% [25], respectively. According to AHA/NHLBI criteria, a significantly higher prevalence of MetS was observed in women than men (55.6% vs. 38.2%) [36].

A case-control study carried out in the United States included women over 65 years (16,323 cases and 100,751 controls). It evaluated the association of MetS using NCEP III criteria and its components with EC. This study found an association between the risk for all EC subtypes and MetS (OR, 1.39), impaired fasting glycemia (OR, 1.36), AH (OR, 1.31), and high TG levels (OR, 1.13) [22].

### Metabolic syndrome components and endometrial cancer

#### Overweight, obesity, and abdominal obesity

Overweight (BMI ≥25 kg/m^2^) and obesity (BMI ≥30 kg/m^2^) are classified based on BMI. However, BMI does not consider factors related to body composition, including the ratio of fat mass to lean mass. For this reason, other measurements such as WC (specific to each population) are more useful in identifying abdominal obesity [36].

A population-based study evaluating the global cancer burden from being overweight and obese found that 63.6% of all endometrial, breast, and colon cancer cases are collectively attributable to high BMI. It is possible that if the current pattern of increasing obesity continues, the prevalence of these cancers will continue to rise [37].

In a UK population-based study that included 5.24 million adult women, they identified that for each 5 kg/m^2^ increase from a BMI ≥25 kg/m^2^, the risk of EC increased 62%. The same study attributed 41% of EC to excess weight [21].

Overweight and obesity are characterized by excess adipose tissue and altered adipose biology [38,39]. Adipose tissue has an almost unlimited lipid storage and expansion capacity. It is a complex endocrine organ responsible for various functions such as energy homeostasis, the regulation of the inflammatory response, and the stimulation of cell proliferation pathways.

The location of adipose tissue is an essential factor, and abdominal obesity is particularly harmful. It is determined by the accumulation of visceral adiposity, characterized by increased fat surrounding the intra-abdominal organs and correlates with WC [38,40]. Excess visceral adiposity in women may lead to an increase in levels of androgen precursors and an increase in aromatase activity, resulting in a significant rise in estrogen levels. In an environment deficient in progesterone, estrogen induces mitogenic effects in the endometrial tissue. The primary mechanism involves the regulation of the cell cycle balance (proliferation, differentiation, and apoptosis). Growth stimulation increases proliferation rates. In cells that have accumulated mutations in proto-oncogenes, this stimulation may ensure the survival of these cells and promote growth and expansion, allowing them to develop as a tumor, which is a vital mechanism relating to EC risk [41].

#### Mechanisms linking adipose tissue inflammation and tumorigenesis

In homeostatic conditions, adipose tissue comprises of adipocytes, stromal cells, and several immune system cell populations that produce mediators and cytokines that maintain a T helper 2 (Th2) environment. Stromal cells, regulatory T cells, and eosinophils produce the cytokines as interleukin 33 and 5 (IL-33 and IL-5), essential for a healthy adipose tissue [42] and the browning of adipose tissue, a critical regulator of metabolic health [40]. This environment contributes to the polarization of the macrophages to a type 2 phenotype (M2) by the production of IL-4 and IL-10 [43]. M2 macrophages sustain an anti-inflammatory environment, by promoting insulin sensitivity and metabolic homeostasis [44]. The adipocytes produce adiponectin, an adipokine with anti-inflammatory activity and a critical glucose and lipid metabolism regulator [45].

The development of obesity involves adipose tissue inflammation. During the process of obesity, adipocytes become hypertrophic, and hyperplastic and increase the production of leptin instead of adiponectin. Leptin is an anorexigenic hormone that directly correlates with white adipose tissue mass [46]. In addition, it is a pro-inflammatory adipokine. Most immune cell populations express the leptin receptor. Its activation leads to the synthesis of inflammatory cytokines such as IL-6, IL-12, IL-18, and tumor necrosis factor-α (TNF-α). It also synthesizes chemokines, such as monocyte chemotactic protein-1 and IL-8, which attract immune cells from circulation, including neutrophils, natural killer (NK) cells, monocytes, and effector lymphocytes [47]. As a result of adipose tissue growth, adipocytes die of necrosis, releasing free fatty acids (FFA), and damage-associated molecular patterns (DAMPs).

These molecules signal resident macrophages to activate, polarize to a type 1 inflammatory phenotype (M1) to phagocytize the dead tissue, and produce inflammatory cytokines and alarmins that attract immune cells from circulation [48]. When immune cells reach the obese adipose tissue, they encounter several inflammatory components, FFA, DAMPs, leukotrienes, TNF-α, IL-1β, and IL-6. This pro-inflammatory environment activates neutrophils, M1 macrophages, and lymphocytes that differentiate to a Th1 phenotype, producing interferon-γ. It further promotes inflammation and activation of the immune response [42].

These changes in immune cell composition and phenotype in the obese adipose tissue promote hormone and metabolic alterations that, together with inflammation, constitute the ideal environment for the development of a tumor [49-54]. One event related to these alterations is that adipose tissue expansion leads to localized hypoxia. The lack of oxygen activates hypoxia-induced factor 1-α that promotes angiogenesis [49], facilitating the access of inflammatory cytokines, and other metabolic components to the circulation. Among these metabolic components, insulin and insulin-like growth factor (IGF) increase in response to the insulin resistance (IR) developed during the inflammatory process and oxidative stress in the obese adipose tissue. Both insulin and IGF bind to insulin receptors (IR-A and IR-B) and IGF receptors (IGF-R) [53], signaling metabolic and mitogenic pathways. IR and IGF-R mediate their effects by activating pathways that ultimately signal PI3K-Akt, mTOR, and MAPK/ERK. The PI3K-Akt pathway is involved in cell survival and regulation of apoptosis, whereas the MAPK/ERK pathway is implicated in cell proliferation. The Akt pathway activates mTOR; this results in protein synthesis, cell growth, and preparation of cells to enter the cell cycle [55,56]. Furthermore, increased levels of insulin and IGF increase aromatase activity that results in an increased production of estrogen. As described above, chronically increased estrogen levels cause endometrial dysplasia and may promote malignant transformation. Inflammatory cytokines such as IL-6 and TNF-α also promote the synthesis of aromatase [57,58]. IL-6 and TNF-α are inflammatory cytokines produced mainly by the innate immune system cell populations, such as macrophages. In the obese adipose tissue, M1 macrophages produce IL-6 and TNF-α.

The effects of these cytokines have been thoroughly described in several tissues and organs, including endothelium, liver, pancreas, hypothalamus, bone marrow, and skeletal muscle. Importantly, they induce the acute phase response in the liver, leading to the production of C-reactive protein (CRP), proteins of the complement system, coagulation factors, and fibrinogen [59]. The signaling of these cytokines receptors leads to the activation of multiple transcription factors, including nuclear factor-kappa B (NF-κB). Nf-kB promotes cell proliferation and survival, increased production of reactive oxygen species (ROS), IR, and angiogenesis [51,53,60]. These mechanisms favor a tumorigenic environment characterized by inflammation, oxidative stress, insensibility to growth inhibitors, self-sufficiency in growth signals, proliferation, evasion of apoptosis, sustained angiogenesis, tissue invasion, and metastasis potential.

**FIGURE 1:**
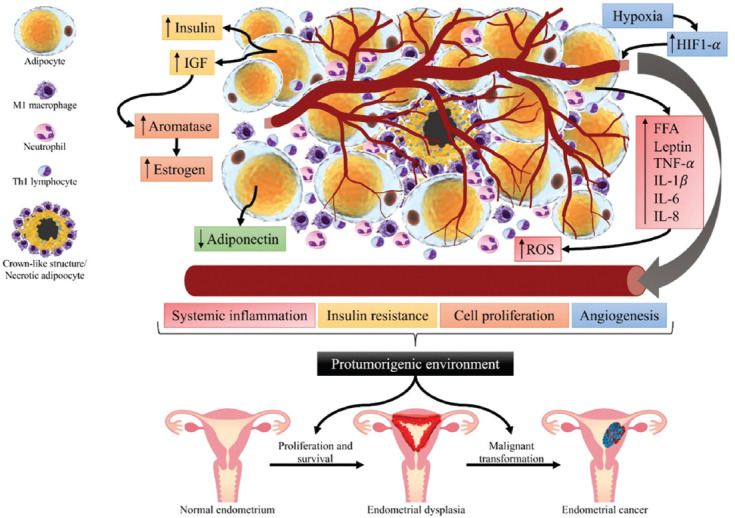
Obesity-derived inflammation and tumorigenic environment may play a role in the development of EC. Obese adipose tissue is characterized by hyperplasia and hypertrophic adipocytes, the formation of crown-like structures, increased secretion of FFA and inflammatory cytokines, and a decreased secretion of adiponectin. The inflammatory mediators attract from circulation neutrophils, Th1 lymphocytes, monocytes that become M1 macrophages, among other cells, all of which contribute to a pro-inflammatory environment that increases the generation of ROS. Hypertrophic adipocytes increase the secretion of insulin and IGF-1 that contribute to insulin resistance and promote cell growth. The hypoxic adipose tissue leads to the production of HIF-1α and angiogenesis. These events create a protumorigenic environment that reaches other tissues and organs, including the endometrium. The increase in aromatase and estrogen is essential in signaling the endometrial tissue to proliferate and develop dysplasia. The high levels of ROS and inflammatory signals may lead to a malignant transformation of the dysplastic tissue, thus developing endometrial cancer. Created by Denisse Castro-Eguiluz. EC: Endometrial cancer; FFA: Free fatty acids; ROS: Reactive oxygen species; IGF-1: Insulin growth factor-1; HIF-1α: Hypoxia-inducible factor-1α.

The obesity-derived inflammation and protumorigenic environment have been proposed to play a role in the development of EC (Figure 1) [57,58]. In addition, to the process previously described, long-term estrogen stimulation is determinant in developing EC [61,62].

The complex interaction between sex steroid hormones, cytokines, and growth factors in the endometrium can lead to a local inflammatory medium that increases estrogen production and inflammatory cytokines, creating an environment that favors tumor initiation and maintenance. Evidence demonstrates an aberrant expression of NF-κB in the nuclei of proliferating endometrium, which may lead to endometrial hyperplasia and eventually EC. Results from the European Prospective Investigation into Cancer and Nutrition (EPIC) cohort also support the role of inflammation in endometrial carcinogenesis [57]. Additionally, to the effects of adipose tissue inflammation, studies have demonstrated that adipose stem cells (ASCs) infiltrate cancer lesions and promote cancer progression. EC cells can fuse with ASCs, which alters extracellular matrix mechanics. ASCs also produce plasminogen activator inhibitor-1, which has a role in invasion and metastasis and is associated with a poor prognosis in EC patients [34,60,61].

#### Alterations in glucose metabolism

T2DM is characterized by elevated insulin levels and IGF-1 levels with hyperglycemia [63]. Evidence supports an independent association between T2DM and an increased risk of EC [64,65]. A meta-analysis that included 25 studies showed that women with EC have higher fasting insulin levels [66].

IR is characterized by reduced sensitivity to insulin in responsive tissues, leading to increased blood insulin and glucose levels [67]. Obesity, physical inactivity, and genetic predisposition can lead to IR [68], the leading cause of T2DM. IR occurs years before it shows clinical signs. This period is known as a prediabetic state and it has an essential role in the development and progression of EC [69,70]. According to the diabetes Atlas of the IDF, the worldwide prevalence of diabetes caused by IR continues to increase [71].

Evidence supports that IR and EC share some risk factors, suggesting that IR and EC may develop simultaneously [11]. On the other hand, IR results in hyperinsulinemia, which triggers physiological effects leading to carcinogenesis because insulin is an important anabolic hormone that stimulates cell proliferation [72].

Insulin exerts its biological effect by binding to specific receptors located on the cell membrane. Once insulin interacts with its receptor and is activated, signaling cascades that depend on an organized number of protein interactions begin, with insulin receptor substrate proteins being essential in the process [73]. Insulin signal transduction involves two isoforms of the insulin receptor. These isoforms are produced through alternative transcriptional splicing. The IR-A recognizes insulin and IGF and has a higher affinity for IGF2 than IGF1. As IR-B is specific for insulin, it is involved in glucose homeostasis [74].

IR-A binds IGF-1 with low affinity, but it has a high affinity for insulin and IGF-2. IGF1R has a high affinity for IGFs. However, blockade of IGF-IR and IR-A in cancer cells does not entirely prevent insulin’s nor IGF’s growth stimulation [61]. In addition, IR-A and IGFR share signaling pathways. However, insulin is involved explicitly in glucose metabolism, and IGFs stimulate cell growth and proliferation [75]. Likewise, the IGF system is known to contribute to human carcinogenesis. IGF2 has been hypothesized to be more closely related than IGF1 to the etiology of EC [76].

It has also been observed that insulin could up-regulate the expression of vascular endothelial growth factor and consequently angiogenesis, a phenomenon observed in the tumor development [56]. Thus, insulin signaling is essential for glucose metabolism [77], and IR may promote tumorigenesis of EC through an indirect pathway or receptor signaling [67].

#### Alterations in lipid metabolism

Alterations in the levels of some serum lipids such as TG [22], total cholesterol [78], low-density lipoprotein (LDL), HDL, and their association with EC have been studied [22].

A prospective population-based study followed 31,473 women for 9 years and found a positive association with high TG levels (age-adjusted hazard ratio, HR, 2.34; and BMI-adjusted HR, 1.79), but not with total cholesterol, LDL, and HDL [79].

A case-control study (942 cases and 1721 controls) found a positive correlation between EC and total cholesterol (OR, 1.62), TG (OR, 1.25), LDL (OR, 1.44), and low HDL (OR, 2.4) [61]. In this study, overweight and obesity strengthened the association between LDL and EC.

Although studies on the mechanisms that explain the association between dyslipidemia and the risk of EC have not been definitive, the following alterations have been identified: activation of fatty acid pathways and increased production of ROS. ROS interact with lipids, proteins, and DNA in cells, causing changes in the integrity of the cell membrane, inducing cell damage, and favoring oncogenesis [61]. On the other hand, increased adiposity may cause the secretion of FFA. These bind to the Toll-like receptor-4, resulting in a sustained inflammatory response, which as aforementioned, contributes to the protumorigenic environment. In addition, excess adiposity increases the aromatase activity and production of estrogen, as described previously [41]. It should be noted that endometrial cells can undergo intense and atypical mitosis both due to estrogenic influx and insulin action, having a double effect: increasing cell proliferation and preventing apoptosis [80]. These mechanisms may lead to dysplasia in endometrial tissue.

#### Arterial Hypertension

The biological mechanisms that explain the relationship between AH and EC are not currently known, and very few studies have explored this relationship. A positive association between AH and EC was demonstrated in a meta-analysis that included 19 case-control studies and 6 cohort studies (RR, 1.61). Authors suggested that AH may promote cellular senescence and apoptosis inhibition, affecting cellular turnover [12,81].

### Endometrial cancer prevention strategies related to metabolic syndrome

Scientific literature has shown that individual, hereditary and environmental factors are involved in the development of EC. It is crucial to consider the role lifestyle plays in developing this cancer, mainly the association between diet and PA, which constitute modifiable factors that play an indispensable role in preventing EC.

#### Diet

Risk modification, above all, due to nutritional factors in the diet, can occur at different stages of the cancer onset process. Several hypotheses have been proposed concerning the mechanisms through which various elements of the diet could contribute to carcinogenesis or prevent it [82].

Carcinogenesis is a process that develops in several steps. These are characterized by molecular changes that cause cellular transformation from normal to malignant cells. Phytochemicals are compounds that exert chemopreventive and anti-inflammatory effects by limiting cytokine secretion, blocking the master regulators of tumor initiation and promotion, reversing the premalignant stage, and inhibiting or slowing tumor progression [83].

Phytochemicals are found in vegetables, fruits, and whole-grain products. They act as antioxidants eliminating free radicals and protecting from the genetic material’s damage. Some are polyphenols, terpenoids, and thiols; their effects have been studied in various diseases such as EC. A group of polyphenols found in soybeans and legumes includes flavonoids and isoflavones, such as genistein, daidzein, and glycitein, which have an antiestrogenic effect in the prevention of EC [84]. A meta-analysis that included epidemiological studies compared a higher intake of isoflavones against a lower intake and found a reduction in the risk of EC [85]. Another polyphenol compound, epigallocatechin gallate, found in green tea, has been associated with prevention of angiogenesis [84].

Zhou et al. evaluated the effect of green and black tea consumption on the risk of EC and found that an increase of one cup per day of green tea was associated with a lower risk of developing EC (RR, 0.89) [86]. Furthermore, coffee contains caffeine, chlorogenic acid, and the terpenoids cafestol and kahweol [87]. A protective effect was found in a dose-response meta-analysis that evaluated the impact of coffee consumption on the risk of EC [88].

Components in foods interact with each other or with other factors (environmental or genetic), and these interactions have physiological effects that are not entirely understood. However, it is known that the diet meets specific characteristics that can mediate estrogen levels and modulate inflammation [87]. The risk of developing EC seems to be associated with a higher intake of foods found in Western diets (pro-inflammatory foods as animal products rich in saturated fatty acids, refined carbohydrates, and ultra-processed foods), [89] which also increase CRP levels [90-92]. Chronic subclinical inflammation can lead to IR [93], stimulating cell proliferation and inhibiting apoptosis [41].

This risk of EC may decrease among women that consume mainly plant-based diets, rich in fruits, vegetables, whole grains, and legumes. This dietary pattern protects against chronic diseases because of its nutrients and bioactive components (vitamins, minerals, fiber, and phytochemicals), which participate in the modulation of inflammation [94]. Also, the micronutrients found in these diets may have a protective effect related to lower amounts of free hormones circulating in the blood [95].

Luvian et al. carried out a systematic review to identify specific functional foods that impacted inflammatory and metabolic mediators. Foods that regulate inflammation include, but are not limited to, specific fruits, whole grain products, low-fat dairy products, green tea, spices, soy foods, nuts and seeds, and particular oils. In addition to the foods described in this study, other available foods may also have inflammatory modulating properties [94].

The diet most studied as a modulator of chronic inflammation is the Mediterranean diet (MedDiet). This diet consists of typical foods of the Mediterranean populations studied in the early 1960s. It is characterized by vegetable products, whole grains, olive oil, vinegar, fish, and foods rich in phenolic compounds (spices and chocolates). The MedDiet has antioxidant and anti-inflammatory characteristics. It is low in saturated fatty acids and simple carbohydrates and rich in vitamins, fiber, unsaturated fatty acids, and antioxidants. This diet includes high amounts of polyphenols and phytochemicals (carotene, lutein, zeaxanthin, lycopene, astaxanthin, phytosterols, or isothiocyanates) [82,96,97]. The health benefits of the MedDiet have been studied in various diseases, including IR, T2DM, cardiovascular disease, nonalcoholic fatty liver disease, dyslipidemia, PCOS, and some types of cancers, including EC [96-98].

Women that adhere to the MedDiet have a lower risk of EC [90,98]. A diet with high consumption of fruits and vegetables, low dietary inflammatory index [90], and low glycemic index [99] confers protection against EC [90]. In contrast, the consumption of ultra-processed foods, which are defined as industrial formulations of food and beverages that contain little or no fresh ingredients, has been linked to obesity [100].

Diets evolve due to various factors and complex interactions. Income, access to food, individual preferences, cultural beliefs, traditions, and geographic, environmental, social, and economic factors all contribute to the complex interaction of food consumption characteristics [101].

Mexican cuisine is a symbol of national identity. In addition, it is one of the most unique, rich, and diverse culinary traditions in the world [102]. However, today it is characterized by the high consumption of ultra-processed foods and sugary drinks, which tend to be energetically dense but poor in nutrients [103]. This westernized dietary pattern can be attributed to the fact that these foods are easily accessible and low in cost. These are displacing the traditional diet, distinguished for being balanced, varied, and rich in nutrients [32]. The change in food culture in Mexico is evident due to health problems in the population: “Urbanization, modernization, and sophistication have frequently led to diets in which a large percentage of energy consumption comes from sugars and fats, which then leads to a higher consumption of salt” [33].

These harmful habits that the population has acquired, accentuating Mexican women, could be contributing to the increase in metabolic diseases and, therefore, a greater risk of developing EC.

In Mexico, more evidence is needed on the benefits of consuming a MedDiet, which may reduce cancer risk. The study by Wu et al. evaluated the intake of fruits and vegetables and its association with cancer risk in Mexican-Americans. Authors found that the intake of fruits and vegetables is associated with decreased cancer risk in this population. They indicated that the increase in consumption of fruits and vegetables could be a promising area for future research as part of a strategy for preventing and controlling cancer among Mexican-Americans, regardless of other risk factors [104].

Likewise, a cross-sectional study by Sahrai et al. evaluated, in Mexican women with breast cancer, associations between dietary patterns defined a priori and anthropometric measures. Authors found that greater adherence to the MedDiet was associated with lower WC and waist-to-hip ratio. Still, no significant association was observed with other dietary patterns [105].

In 2017, the Secretary of Health in Mexico proposed the model of “The Milpa Diet,” whose origins are ancient in our country and strengthened by highlighting its nutritional contributions. Aim is to define and disseminate a model of healthy eating based on Mesoamerican foods that are part of our cultural identity, with a positive impact on health [106].

The “Milpa Diet” consists of the traditional Mexican diet based on the milpa, an agro-food production system developed by the Mesoamerican population [107]. This type of diet mainly includes corn, beans, chili peppers, and squash, in addition to other vegetables rich in fiber, protein, antioxidants, and micronutrients (e.g., nopales, quelites, quintoniles, purslane, green beans, romeritos, huazontle, tomatoes, tomatillos, chili peppers, chayote squash, huitlacoche, watercress, mushrooms, legumes, fresh seasonal fruits, avocados, amaranth, sweet potatoes, fish, shellfish, eggs, and edible insects). The Milpa diet avoids red meat, flour, refined sugars, saturated fats, and ultra-processed foods. One of the essential characteristics of the Milpa diet is the high content of fiber. The benefit of fiber-rich diets has been widely studied and is closely related to the microbiota. Studies have described the role of fiber on the diversity and enrichment of beneficial bacterial species. These play an essential role in the prevention of MetS and cancer. The biological mechanism involves the anti-inflammatory and regulatory effect on immune cell populations. In addition, bacterial fermentation of fiber results in the production of short-chain fatty acids, which provide energy to colonocytes and immune system cells and exert systemic effects that lead to metabolic health (including insulin sensitivity) [108]. These beneficial characteristics are nutritionally similar to the MedDiet’s biocompatibility, cultural suitability, and sustainability [106]. Preferably, this diet should be based on traditional foods and complemented with the other foods from other areas of the world [109].

For prevention strategies, women need to adopt a healthy eating pattern within their cultural context and access to food. For this reason, the “Milpa Diet” is a feasible proposal to prevent EC in the Mexican population.

#### Physical Activity

Regular physical activity (PA) is a guideline used in conjunction with an adequate diet to prevent and reduce obesity and other risk factors associated with EC. Regular PA exerts different health benefits and may also prevent EC [41,110]. The biologic mechanisms that explain the effect of physical exercise in risk reduction of EC have been hypothesized. Several pathways may occur simultaneously with an added effect that contributes to cancer risk reduction. It is well known that the increase in PA leads to a decrease in adiposity and may reduce sedentary behavior. Physiologically, PA increases metabolic function by improving insulin sensitivity, lowering levels of IGF-1, and reducing glucose levels. The decrease in adiposity is associated with a reduction in the secretion of pro-inflammatory cytokines (IL-6, TNF-α, and IL-1β) and an increase in anti-inflammatory mediators (IL-10 and adiponectin). In addition, the decrease in adipose tissue leads to a reduction in the synthesis of androgen precursors and consequently a decrease in estrogen levels [111,112]. Other studies have described the effect of exercise on 5’-AMP-activated protein kinase (AMPK), a signaling molecule with direct effects on transcriptional regulators that inhibit IR and regulate cell growth and survival. Exercise-related increase in AMPK activity may therefore inhibit cancer growth [113]. Together, these mechanisms reduce the risk of EC.

Furthermore, some studies analyze the association of sedentary lifestyle with the risk of developing EC. Gierach et al. measured the hours a person remains in a sitting position among women who exercised three or more times per week or those who exercised less and found that amount of time sitting, or reclining was associated with increased risk of EC [114]. Specifically, sedentary women who spent nine or more hours sitting had twice the risk of EC than more physically active women who sat less than three hours per day (RR, 2.14, 95% CI 1.48-3.10). Furthermore, Moore et al. determined, based on scientific findings, that prolonged sitting, regardless of the amount of moderate or vigorous PA [115], increases the risk of obesity, IR [116,117], premature mortality [118], and EC [119,120].

Wu et al. conducted a study to determine the association between PA and the risk of MetS in Mexican Americans. The moderate and vigorous physical exercise was evaluated. They found that performing PA at moderate intensity for 30 minutes at least 5 days a week or doing vigorous exercise at least 3 days a week for 20 minutes (such as walking, cycling, and running) are associated with the prevention and control of MetS. Thus, PA may be a promising strategy to help prevent EC [121].

Even recreational walks, brief activities in the workplace (such as taking stairs instead of the elevator), PA at home, active play with children, or gardening also contribute to the daily amount needed to improve health. However, it is still necessary to perform them longer [122].

Studies are needed to focus on further supporting dietary recommendations and lifestyle modifications that must be customized according to food availability, geographic location, and health status [123].

## FUTURE DIRECTIONS

The link between microbiota, obesity, and EC has been studied. Preliminary data in murine models reveal that the fecal microbiota was different in lean versus obese mice. The uterine microbiota was different according to the obesity status [124,125]. The microbiota composition has been compared among women of various races and EC. The microbiota profiles identified in murine models with EC overlap, so it would be necessary to carry out further studies to understand better the interrelation of obesity in the microbiota of women with EC [125].

## CONCLUSION

This review focused on analyzing and integrating the information comprised to raise awareness amongst health professionals on the relationship between MetS and EC and the importance of developing prevention strategies. The protumorigenic inflammatory microenvironment has explained this relationship in these metabolic alterations. We propose that the primary prevention strategies for EC related to MetS are lifestyle modifications. These include adopting a diet rich in fruits, vegetables, whole grains, and legumes, according to the individual’s accessibility to food, and avoiding consumption of pro-inflammatory foods. In addition to these are daily PA and avoiding a sedetary lifestyle. Therefore, randomized clinical trials are needed to demonstrate the effect that lifestyle interventions have on the treatment of MetS and the prevention of EC. We also urge the importance of screening for MetS and its components, mainly central adiposity, in all women for early treatment and possibly prevention of EC.

The main limitation of this study is that this is not a systematic review. In addition, we did not find sufficient original articles describing this association of MetS and EC in the Mexican population. This lack of information is a concern because there has been an important increase in the incidence of EC in Mexican women, and we believe that it is associated with an increase in MetS. Further studies are needed to describe this phenomenon in our population.
